# Transforming Cancer Classification: The Role of Advanced Gene Selection

**DOI:** 10.3390/diagnostics14232632

**Published:** 2024-11-22

**Authors:** Abrar Yaqoob, Mushtaq Ahmad Mir, G. V. V. Jagannadha Rao, Ghanshyam G. Tejani

**Affiliations:** 1 School of Advanced Science and Language, VIT Bhopal University, Kothrikalan, Sehore, Bhopal 466114, India; abrar.yaqoob@kalingauniversity.ac.in; 2 Department of Clinical Laboratory Sciences, College of Applied Medical Sciences, King Khalid University, Abha 61421, Saudi Arabia; 3 Department of Mathematics, Kalinga University, Raipur 492001, India; dr.gvvj.rao@kalingauniversity.ac.in; 4 Department of Industrial Engineering and Management, Yuan Ze University, Taoyuan City 320315, Taiwan; p.shyam23@gmail.com; 5 Jadara Research Center, Jadara University, Irbid 21110, Jordan

**Keywords:** cancer classification, Mutual Information, Particle Swarm Optimization, Support Vector Machine

## Abstract

**Background/Objectives:** Accurate classification in cancer research is vital for devising effective treatment strategies. Precise cancer classification depends significantly on selecting the most informative genes from high-dimensional datasets, a task made complex by the extensive data involved. This study introduces the Two-stage MI-PSA Gene Selection algorithm, a novel approach designed to enhance cancer classification accuracy through robust gene selection methods. **Methods:** The proposed method integrates Mutual Information (MI) and Particle Swarm Optimization (PSO) for gene selection. In the first stage, MI acts as an initial filter, identifying genes rich in cancer-related information. In the second stage, PSO refines this selection to pinpoint an optimal subset of genes for accurate classification. **Results:** The experimental findings reveal that the MI-PSA method achieves a best classification accuracy of 99.01% with a selected subset of 19 genes, substantially outperforming the MI and SVM methods, which attain best accuracies of 93.44% and 91.26%, respectively, for the same gene count. Furthermore, MI-PSA demonstrates superior performance in terms of average and worst-case accuracy, underscoring its robustness and reliability. **Conclusions:** The MI-PSA algorithm presents a powerful approach for identifying critical genes essential for precise cancer classification, advancing both our understanding and management of this complex disease.

## 1. Introduction

Human genetic data can significantly enhance the detection and classification of diseases like cancer, with microarray analysis being one of the most precise methods available. This technique structures data into a matrix, where rows represent samples and columns denote genes, often resulting in thousands of genes but only a few hundred samples. This imbalance can lead to high computational costs and challenges in generalizing classifications, while irrelevant genes may introduce “background noise,” obscuring the impact of biologically relevant genes [1]. To combat these issues, current research aims to improve classification accuracy by adopting innovative strategies, reducing dataset size, and eliminating unnecessary noise and redundant information [2,3]. Researchers are increasingly exploring meta-innovative optimization algorithms due to the growing complexity of data dimensions, as the challenge of feature selection and classification in microarray data represents a difficult non-polynomial optimization problem. Successfully identifying unique genes and enhancing classifier performance heavily relies on employing suitable optimization techniques [4]. Consequently, many studies focus primarily on improving classification accuracy, while also considering objectives like minimizing the number of features and removing redundancy. Multi-objective optimization methods can significantly enhance classifier effectiveness by addressing both feature reduction and redundancy issues [5,6,7,8].

In this study, we present a novel hybrid two-stage gene selection algorithm designed to enhance cancer classification accuracy by combining Particle Swarm Optimization (PSO) with the Mutual Information (MI) measure. Our proposed MI-PSO algorithm operates in two distinct stages to systematically identify the most relevant genes for classification. The first stage employs Mutual Information to select genes with high relevance to cancer classification. In the second stage, Particle Swarm Optimization (PSO) refines this selection to identify the most promising subset of genes from the initial candidates. To further improve classification accuracy, we use a Support Vector Machine (SVM) classifier, known for its effectiveness in handling complex datasets, to accurately classify the cancer samples. This hybrid approach leverages the complementary strengths of PSO and MI to achieve superior performance in cancer classification tasks [9,10,11].

### 1.1. Paper Organization

The remaining sections of this paper are organized as follows: Section 2 outlines the proposed algorithms for gene selection and classification, offering a detailed explanation of their methodologies. In Section 3, we present the experimental outcomes derived from applying these algorithms to three distinct microarray datasets, along with the insights garnered from the analyses. Furthermore, Section 4 delves into a thorough discussion of the experimental results, elucidating their implications and significance. Finally, Section 5 offers final thoughts, encapsulating the study’s results and proposing possible avenues for future research in this area [12].

### 1.2. Motivation for This Study

Accurate cancer classification is a cornerstone of effective treatment planning and personalized medicine. However, the high dimensionality and complexity of gene expression data pose significant challenges in identifying the most relevant genetic markers for precise diagnosis and prognosis. Traditional gene selection methods often struggle to balance accuracy and computational efficiency, leading to suboptimal classification outcomes [13].

The motivation behind developing the Two-stage MI-PSA Gene Selection algorithm is to address these challenges by providing a robust and efficient solution for gene selection and cancer classification. By leveraging the strengths of Mutual Information (MI) for preliminary filtering and Particle Swarm Optimization (PSO) for refining the gene set, this novel approach aims to enhance classification accuracy and provide deeper insights into the genetic underpinnings of cancer.

This innovative method enables researchers and clinicians to identify key genes more effectively, aiding early diagnosis, deeper insights into cancer biology, and targeted therapy development. MI-PSA’s success with the breast cancer dataset highlights its potential as a valuable tool in cancer research, encouraging broader exploration in various oncological settings.

## 2. Related Work

In this section, we review the existing literature on breast cancer classification and diagnosis, focusing on recent advancements in deep learning and machine learning methodologies. We explore various techniques and models that have been proposed to enhance the accuracy and efficiency of breast cancer detection, including feature extraction methods, ensemble learning strategies, and the application of transfer learning. By synthesizing these studies, we aim to highlight the progress made in the field and identify potential gaps that our research seeks to address. Hanan et al. present a computer-aided diagnosis method for breast cancer classification using deep neural networks (ResNet 18, ShuffleNet, Inception-V3Net) and transfer learning. The method achieves binary classification accuracies of 99.7%, 97.66%, and 96.94%, and multi-class accuracies of 97.81%, 96.07%, and 95.79%, respectively [14]. Moloud Abdar et al. introduced two uncertainty quantification methods, DE and EMC, to analyze skin cancer datasets and reduce overconfidence in diagnoses. The final solution achieved accuracies of 88.95% and 90.96% and F1-scores of 89.00% and 91.00% for the first and second datasets, respectively, demonstrating the effectiveness of their TWDBDL model in medical image analysis [15]. Mahmoud Ragab et al. developed the EDLCDS-BCDC for breast cancer diagnosis using ultrasound images. The method preprocesses images, segments them with the Chaotic Krill Herd Algorithm, extracts features using VGG-16, VGG-19, and SqueezeNet, and classifies them with Cat Swarm Optimization and a Multilayer Perceptron. Simulations demonstrate its superior performance over recent methods [16]. Ruxandra Stoean introduces a method for tuning CNN convolutional layers and ranking hyperparameter importance using surrogate models: random forests (RF), support vector machines (SVM), and Kriging. Convolutional configurations are generated through Latin hypercube sampling, with accuracy derived from real CNN runs. The approach evaluates hyperparameter fitness, with RF offering implicit selection, SVM enhanced by an additional algorithm, and Kriging providing rankings. The method is tested on histopathological image interpretation for colorectal cancer diagnosis [17]. Shallu Sharma et al. developed two machine learning approaches for multi-classification of breast cancer in the BreakHis dataset. One approach uses handcrafted features (Hu moments, color histograms, Haralick textures), while the other employs transfer learning with pre-trained networks (VGG16, VGG19, ResNet50). The transfer learning approach outperformed the handcrafted method, with VGG16 and linear SVM achieving the highest accuracies: 93.97% (patch-based) and 93.25% (patient-based) for 40× magnification. “Fibro-adenoma” (benign) and “Mucous Carcinoma” (malignant) were the most complex classes [18]. Devakishan Adla et al. proposed the DLCAL-SLDC, an automated deep learning model for skin lesion detection and classification using dermoscopic images. The model employs hair removal and noise reduction during pre-processing, followed by Tsallis entropy-based segmentation to identify lesions. Features are extracted using a Capsule Network with a class attention layer and the Adagrad optimizer. Classification is performed using a Swallow Swarm Optimization-based Convolutional Sparse Autoencoder. Validated on the ISIC dataset, the DLCAL-SLDC achieved 98.50% accuracy, 94.5% sensitivity, and 99.1% specificity, outperforming other methods [19]. Maad M. Mijwil developed a deep learning network using a convolutional neural network (ConvNet) model to analyze over 24,000 skin cancer images with three architectures: InceptionV3, ResNet, and VGG19. The high-resolution images were sourced from the ISIC archive (2019–2020). Among the tested architectures, InceptionV3 emerged as the best, achieving a diagnostic accuracy of approximately 86.90%, precision of 87.47%, sensitivity of 86.14%, and specificity of 87.66% [20]. Nonita Sharma et al. propose a snapshot ensembling technique to create an efficient model for disease diagnosis. Using t-SNE for enhanced scatter plots, the model integrates predictions from various base models to improve accuracy. Applied to the Wisconsin Breast Cancer Dataset (WBCD), it achieved 86.6% accuracy, outperforming state-of-the-art models like averaging (81%) and stacked ensemble (84.7%), showcasing its effectiveness [21]. Lei Cui et al. developed a survival analysis system using deep learning, consisting of three components: (1) A cellular feature learning module with a deep neural network and global average pooling that aggregates biologically relevant information into patient-level feature vectors. (2) A Cox proportional hazards model with an elastic net penalty for robust feature selection. (3) A biomarker interpretation module that identifies key image regions influencing the model’s decisions. The system demonstrated strong predictive power on The Cancer Genome Atlas lung cancer dataset, assessed using the log-rank test and concordance index [22].

## 3. Materials and Methods

### 3.1. Proposed Cancer Classification Approach

In our proposed cancer classification approach, the selection of genes crucially determines the classification of cancer samples. We employ a two-stage method: initially using Mutual Information (MI) for feature selection to identify informative features, followed by Particle Swarm Optimization (PSO) to optimize these features. Subsequently, a Support Vector Machine (SVM) classifier utilizes hyperplanes to classify each sample in the microarray data, where “0” represents “No Cancer” and “1” represents “Cancer”. The gene subset identified by the MI-PSO method is further processed using Support Vector Machine (SVM) with different kernel functions. Analyzing various microarray cancer datasets presents several challenges, as using a single kernel function may not effectively capture all patterns. To enhance classification adaptability across various microarray datasets, multiple kernel functions are employed. The optimal kernel function for the cancer microarray data is determined and chosen for classification, ensuring enhanced suitability and adaptability in the analysis process [23,24,25,26].

### 3.2. Selecting Informative Genes Using Mutual Information

In probability theory, Mutual Information measures the relationship between two random variables by evaluating how much knowledge of one variable reveals about the other within a shared context. Take, for instance, a system involving two discrete random variables, X and Y, where X is the input variable with N_X_ possible values (x ∈ X), and Y is the output variable with N_Y_ possible values (y ∈Y). Mutual Information, denoted as I(X:Y), reflects the extent to which uncertainty in one variable is reduced upon knowing the other variable’s value. This metric can be expressed in terms of entropy and conditional entropy, where entropy represents the uncertainty associated with a single random variable, and conditional entropy reflects the uncertainty remaining in one variable after the other variable’s value is known. The computation of Mutual Information provides important insights into the relationship between the variables aiding in various statistical analyses and information-theoretic applications [27].

(1) $$I(X:Y)=H(Y)-H(\frac{Y}{X})$$

In this context, H(Y) represents the uncertainty level in the output variable Y, as defined in Equation (2), while H(Y∣X) denotes the remaining uncertainty in Y when the input variable X is known, as defined in Equation (3). The difference between these two quantities reflects the amount of information gained about the output variable Y when the input variable X is known.

(2) $$H\left(Y\right)=-\sum_{J=1}^{Ny}P(y_{j})\times \log[P\left(y_{j}\right)]$$

In this context, P(yj) represents the probability of event Y occurring with outcome yj, while P(xi) signifies the likelihood of event X occurring with outcome xi within the provided dataset. Additionally, P ($Y_{J}/X_{I}$) refers to the conditional probability, indicating the likelihood of event Y resulting in outcome yj when event X occurs with outcome xi [28].

(3) $$H(X\backslash Y)=-\sum_{J=1}^{Nx}P\left(x_{i}\right)[\sum_{J=1}^{Ny}P(Y_{J}/X_{I})\times \log[P(Y_{J}/X_{I})]$$

Higher mutual information indicates a better understanding of the output variable and less uncertainty. In microarray cancer data, genes with greater mutual information provide more significant insights, leading to more accurate classification. These genes possess enhanced discriminatory power, enabling them to effectively distinguish between different classes of cancer samples. As such, prioritizing genes with higher mutual information enhances the precision and dependability of classification models, ultimately contributing to improved cancer diagnosis and treatment strategies [29].

In this approach, MI is used to evaluate the statistical dependency between each gene and the target class (cancerous or non-cancerous). The higher the MI value, the stronger the relationship between a gene and the class label.

The filtering process begins with the calculation of MI values for each gene in the dataset. These values are used to rank genes based on their relevance to the target variable. To ensure that only significant features are selected, genes with higher MI values are retained, while those with lower MI values, which exhibit redundancy or low relevance, are discarded. The selection process also includes the identification of redundant features by analyzing the correlation between the genes. Genes that are highly correlated with one another are removed to avoid multicollinearity, thus ensuring that the selected subset is both informative and non-redundant.

The formal implementation of this MI filtering process is outlined as follows:

1. MI Calculation: For each gene in the dataset, calculate the MI between the gene and the target class (cancerous or non-cancerous).
2. Gene Ranking: Rank genes in descending order based on their MI values. Higher MI values indicate stronger relevance to the target class.
3. Feature Selection: Select the top genes based on their MI ranking. The number of selected genes is determined by the desired level of dimensionality reduction.
4. Redundancy Removal: Remove genes that are highly correlated with others to avoid redundancy in the selected gene subset.

This filtering process reduces the dimensionality of the dataset by selecting the most relevant and non-redundant features, thus setting the stage for the subsequent optimization and classification steps.

**Figure 1:**
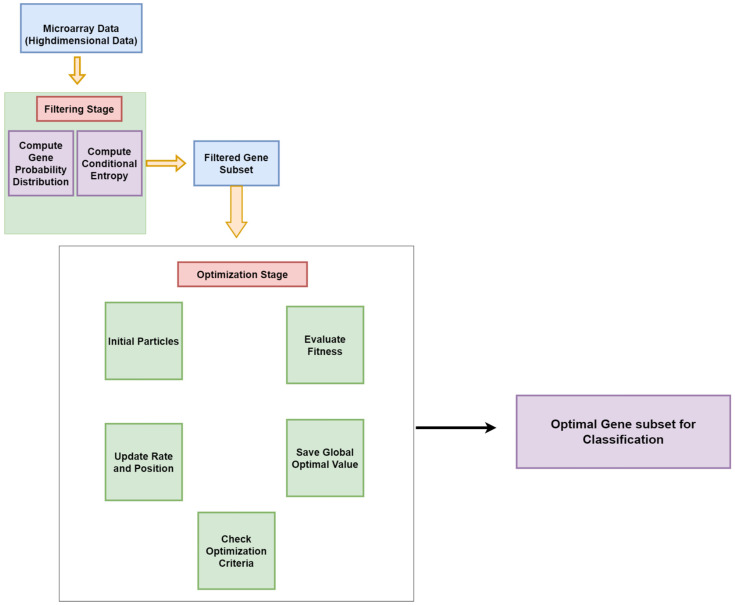
Flowchart of gene selection stage.

Figure 1 illustrates the process flow for the two-stage MI-PSO gene selection method. In the initial stage, key features are identified based on their Mutual Information values, serving as a filtering method to select genes with high information gain relevant to the cancer class. These selected genes are then input into the Particle Swarm Optimization (PSO) process, which acts as a wrapper method to refine and determine the most optimal gene subset. By eliminating redundant and irrelevant genes in the first stage, the final gene subset from the second stage comprises only the most informative genes, thereby improving classification effectiveness. The proposed approach has two main objectives: first, to identify the optimal gene subset from various microarray cancer datasets, and second, to achieve the precise classification of cancer samples using this selected gene subset, thereby improving classification accuracy across different microarray cancer datasets. This method addresses the challenges posed by the variability and complexity of cancer data by efficiently selecting relevant genes to improve cancer sample classification accuracy. After completing the hybrid two-stage gene selection algorithm, the resulting gene subset is passed on to the final classification stage, which employs the MI-PSO-based gene selection method. This final stage culminates in accurately classifying cancer samples using the identified gene subset. The subsequent subsections provide detailed insights into the MI-PSO-based gene selection approach, elaborating on its methodologies and procedures, and highlighting its contributions to more effective cancer diagnosis and treatment strategies. Figure 1 below shows the process flow of gene selection stage.

### 3.3. Particle Swarm Optimization (PSO)-Based Feature Selection

Particle Swarm Optimization (PSO) is an optimization technique inspired by the social dynamics of bird flocks and fish schools. In PSO, particles explore a search space to locate an optimal solution, adjusting their positions and velocities based on their own experience and that of neighboring particles. The velocity update (Equation (4)) considers each particle’s current velocity, its distance from its best-known position, and its proximity to the global best position:

(4) $$v_{i}\left(t+1\right)=w.v_{i}\left(t\right)+c_{1}.r_{1}.\left(p_{i}\left(t\right)-x_{i}\left(t\right)\right)+c_{2}.r_{2}.\left(g\left(t\right)-x_{i}\left(t\right)\right)$$

where $v_{i}\left(t\right)$ is the velocity of particle i at iteration t, w represents the inertia weight, ${C_{1}\;and\;C}_{2}$ are the coefficients for acceleration, $r_{1}\;and\;r_{2}\;are\;random\;values$ sampled from a uniform distribution, $p_{i}\left(t\right)$ is the most favorable position discovered by the particle i, $x_{i}\left(t\right)$ is the present location of the particle i, and $g\left(t\right)$ is the best position found globally [30]. After updating the velocities, the particle positions are updated accordingly in Equation (5):

(5) $$x_{i}\left(t+1\right)=x_{i}\left(t\right)+v_{i}\left(t+1\right)$$

Each particle evaluates its fitness and updates its best-known position if a better solution is found by using Equation (6):

(6) $$p_{i}\left(t+1\right)=argmin(fx_{i}\left(t+1\right),fp_{i}\left(t)\right)$$

The swarm also updates its global best position based on the best-known positions of all particles using Equation (7):

(7) $$g\left(t+1\right)=argmin(f(p_{i}\left(t+1\right),f(g\left(t)))\right)$$

SO terminates when a predetermined stopping condition is met, such as reaching a maximum number of iterations or achieving the desired level of solution quality. Parameters like the number of particles, inertia weight, and acceleration coefficients must be set beforehand prior to executing the algorithm. Through this iterative process of exploration and exploitation, PSO effectively converges towards an optimal solution [31].

Particle Representation in Feature Selection

In the context of feature selection, each particle in the PSO swarm represents a potential subset of features. The position of a particle, $x_{i}(t)$, is encoded as a binary or real-valued vector, where each dimension corresponds to a specific feature in the dataset. For binary encoding, $x_{i}(t)$ can be {0,1}, indicating whether a feature is excluded (0) or included (1) in the selected subset. In the case of real-valued encoding, a threshold is applied to determine inclusion or exclusion.

Objective Function for Fitness Evaluation

The fitness of a particle, *f* ($x_{i}(t)$), is evaluated based on the classification performance (e.g., accuracy, F1-score) of a machine learning model trained on the selected subset of features. Alternatively, fitness can incorporate criteria like minimizing the subset size while maximizing classification performance. For instance:

$$f(x_{i}(t))=\alpha .Error(\left(x_{i}\left(t\right)\right)+\beta \frac{\left|x_{i}(t)\right|}{D}$$

where Error $x_{i}(t)$ is the classification error, $\left|x_{i}(t)\right|$ is the number of selected features, D is the total number of features, and α, β are weight parameters balancing classification performance and subset size.

Velocity and Position Updates for Feature Selection

The velocity update equation adapts to feature selection by considering the search space constraints. In binary encoding, velocities $v_{i}(t+1)$ are transformed into probabilities using a sigmoid or similar activation function, and positions $x_{i}\left(t+1\right)$ are updated based on these probabilities:

$$x_{i}\left(t+1\right)=\left\{\begin{array}{c} 1\;if\;rand\left(\;\right)<sigmoid(v_{i}\left(t+1\right))\\ 0\;\;\;\;\;\;\;\;otherwise \end{array}\right.$$

Connection Between MI Filtering, PSO, and SVM

To clarify the interconnections between MI filtering, Particle Swarm Optimization (PSO), and Support Vector Machine (SVM), we have expanded on how these techniques are integrated to form a cohesive workflow for gene selection and classification.

### 3.4. Classification Based on Support Vector Machine

Support Vector Machine (SVM) is a robust supervised learning algorithm commonly applied in classification and regression tasks. SVM’s primary objective is to determine the optimal hyperplane that effectively separates data points belonging to different classes within a high-dimensional space. Given a set of training data consisting of input–output pairs ($x_{i},y_{i})\;where\;x_{i}$ represents the input feature and $y_{i}$ is the corresponding class label with $y_{i}$∈{−1,+1} for binary classification. SVM aims to discover the hyperplane characterized by w⋅x + b = 0. Here, w signifies the weight vector, and b represents the bias term. The goal is to maximize the margin, which is the distance between the hyperplane and the closest data points of each class. This is formulated as an optimization problem [32]:

(8) $$\mathrm{Minimize}\;\frac{1}{2}\;{\left||w|\right|}^{2}$$

Subject to the constraints

(9) $$y_{i}\left(w\cdot x+b\right)\geq 1\;for\;i=1,2,3\ldots n$$

When the data cannot be separated linearly, SVM employs a kernel trick to map the input features into a higher-dimensional space, allowing for the identification of a linear decision boundary. The kernel function K($x_{i},y_{i})$ calculates the dot product of the mapped feature vectors. Commonly used kernel functions include the linear kernel K($x_{i},y_{i})$ = ($x_{i},y_{i})$, polynomial kernel (K($x_{i},y_{i})$ = (ℽ($x_{i}.y_{i})$ + r)^d^), and radial basis function kernel K($x_{i},y_{i})$= ${e^{-ℽ||x_{i}-y_{i}||}}^{2}$. The selection of the kernel function impacts both the decision boundary and the classification accuracy of the SVM. By optimizing this process, SVM efficiently learns a boundary that maximizes the separation between classes, establishing it as a versatile and widely adopted tool in machine learning [33,34,35]. Table 1 displays the parameter configuration of the proposed hybrid method. Algorithm 1 outlines the pseudocode for the proposed method, which combines Mutual Information (MI)-based gene selection and Particle Swarm Optimization (PSO) to identify informative genes crucial for accurate cancer classification using Support Vector Machine (SVM) as the classifier.
**Algorithm 1:** Pseudocode for the proposed methodfunction MI_PSA_ Gene_ Selection (Data, Labels):    // Stage 1: Mutual Information (MI) based gene selection    selected_ genes = MI_ Selection (Data, Labels)    // Stage 2: Particle Swarm Optimization (PSO) refinement    best_ gene_ set = PSO_ Refinement (Data [selected_ genes], Labels)    return best_ gene_ set function MI_ Selection (Data, Labels):    // Calculate mutual information between each gene and class labels    mutual_ information_ scores = calculate _ mutual_ information (Data, Labels)    // Select top genes based on mutual information scores    selected_ genes = select _top _genes (mutual _ information _scores)    return selected_ genes function PSO_ Refinement (Data, Labels):    // Initialize particle swarm    particles = initialize_ particles ()    global_ best_ position = null    // PSO optimization loop    while not convergence _criteria _met ():        for particle in particles:            // Evaluate fitness of particle’s gene selection            fitness = evaluate _fitness (particle. position, Data, Labels)            // Update particle’s best position and global best position            if fitness > particle. best_ fitness:                particle. best_ position = particle. position                particle. best_ fitness = fitness            if fitness > global_ best_ fitness:                global_ best_ position = particle. position                global_ best_ fitness = fitness        // Update particle positions using velocity and global best position        update_ particle_ positions (particles, global_ best_ position)    return global_ best_ position

### 3.5. Experimental Setup and Data Structure

In this study, we used Ubuntu 20.04.5 LTS with WSL and Visual Studio Code (VS Code) 1.82.0 for coding in Python 3.11.0. Leave-One-Out Cross-Validation (LOOCV) was applied for model evaluation. Experiments were conducted on a high-performance system with an Intel Core i9-12900k processor, 64 GB RAM, and an Nvidia RTX Quadro A5000 GPU. Detailed characteristics and specifics of these datasets are listed in Table 2. The dataset, detailed in Table 2 formed the foundation of our research on gene selection from a high-dimensional biological dataset, which can be accessed at https://csse.szu.edu.cn/staff/zhuzx/Datasets.html, accessed on 19 September 2024.

## 4. Results and Discussion

Table 3 presents the performance assessment of the proposed approach alongside two established techniques, Mutual Information and Support Vector Machine (SVM), in the task of selecting genes, likely for predictive modeling or classification purposes in a biological context. Each row corresponds to a different scenario where a specific set of genes is chosen for analysis ranging from 13 to 47. The accuracy metrics provided in the table offer insights into how well each method performs across various scenarios, with values provided for the best, average, and worst cases. These metrics serve as indicators of the dependability and efficiency of each method in selecting genes that substantially contribute to the predictive accuracy or classification accuracy of the model. Examining the results, it is evident that the proposed method consistently outperforms or matches the performance of Mutual Information and SVM across different numbers of selected genes. This superiority is particularly notable in scenarios involving larger gene sets, where the proposed method maintains higher accuracy metrics even in the worst-case scenarios. For instance, when selecting 19 genes, the proposed approach achieves a level of accuracy of 99.01% in the best case, surpassing both Mutual Information (93.44%) and SVM (91.26%). This trend continues across various gene selection scenarios, with the proposed method consistently demonstrating superior accuracy, especially in more challenging situations where another method falters. Moreover, the proposed method’s robustness is highlighted by its ability to maintain high accuracy across different scenarios, whereas Mutual Information and SVM exhibit more variability in their performance. This suggests that the proposed method may offer more reliable and consistent results, making it a promising choice for gene selection tasks in practical applications. Table 3 presents the classification accuracy of proposed method and other algorithms individually.

When comparing the different gene selection methods, the proposed approach consistently demonstrates strong performance, particularly in achieving high accuracy across various scenarios. Further validation and testing may be necessary to confirm these findings and assess the method’s applicability to specific biological datasets and research objectives.

### 4.1. Confusion Matrix

**Figure 2:**
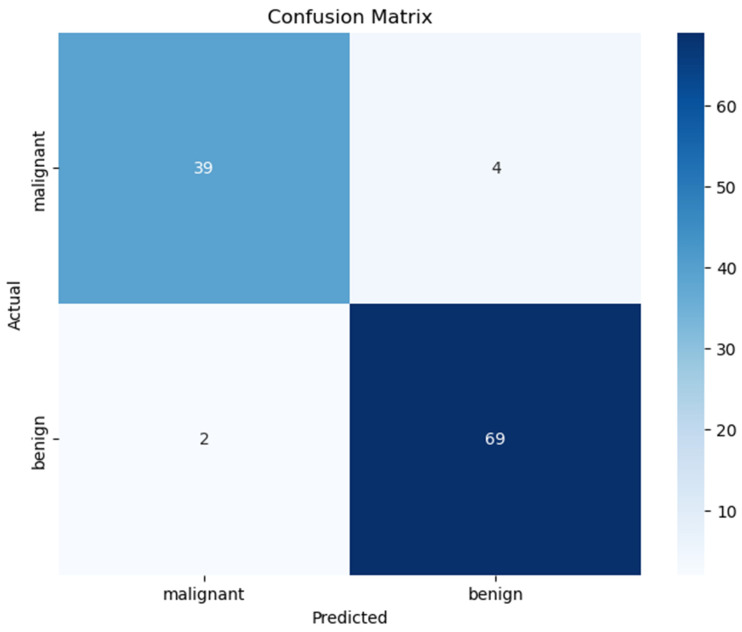
Confusion matrix based on the breast cancer data set using proposed method.

Figure 2 presents a key metric used to evaluate classification model performance: the confusion matrix. This tool provides a comprehensive view of the model’s accuracy by comparing its predictions with actual outcomes in a grid format. The matrix consists of four main elements: true positives (TPs), true negatives (TNs), false positives (FPs), and false negatives (FNs). True positives are cases correctly predicted as positive, while true negatives are those correctly predicted as negative. False positives occur when negative instances are incorrectly predicted as positive, and false negatives happen when positive instances are misclassified as negative. This matrix offers valuable insights into the model’s performance, helping researchers understand its strengths and identify areas for improvement.

The confusion matrix is a valuable tool for assessing a model’s performance, revealing its strengths and weaknesses. By examining its components—true positives, true negatives, false positives, and false negatives—researchers can compute key metrics like accuracy, precision, recall, and F1-score. These metrics offer a thorough evaluation of the model’s classification ability. Insights from the confusion matrix help refine models and guide decisions on their practical use. Equations (10)–(13) describe how these metrics are calculated from the matrix data.

(10) $$ACC=\frac{TP+TN}{TP+TN+FP+FN}$$

(11) $$P=\frac{TP}{TP+FP}$$

(12) $$Sn=\frac{TP}{TP+FN}$$

(13) $$F-score=2\times \frac{P\times Sn}{P+Sn}$$

### 4.2. Precision Recall Curve

**Figure 3:**
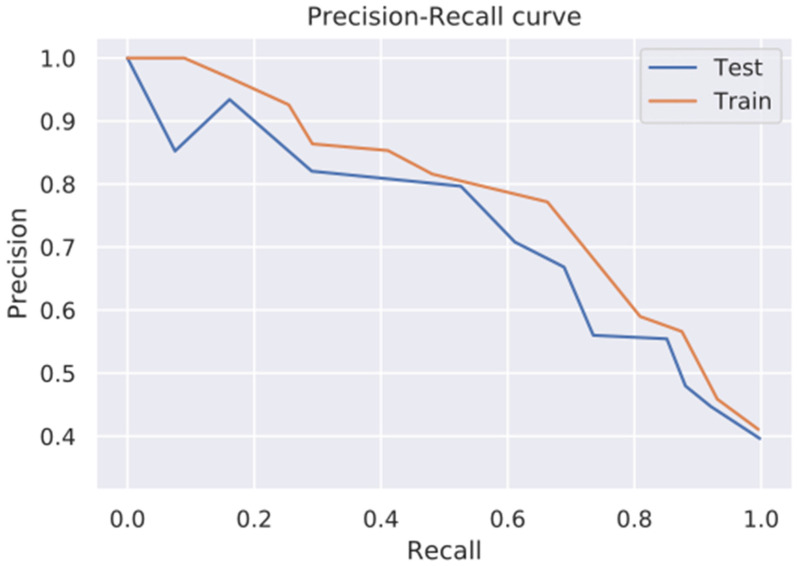
Precision–recall curves.

To strengthen its results, this study includes key visual components, notably, Figure 3, which displays the precision–recall (PR) curve. This tool provides a detailed analysis of the proposed method’s performance by illustrating the balance between precision (the accuracy of positive predictions) and recall (the proportion of true positives correctly identified). The PR curve offers researchers valuable insights into the method’s effectiveness, facilitating a comprehensive assessment of its overall performance.

### 4.3. Area Under the Curve

A high Area Under the Curve (AUC) in Receiver Operating Characteristic (ROC) curves reflects strong model performance. These curves showcase the balance between sensitivity (true positive rate) and the false positive rate, providing insights into classification accuracy at various thresholds. Furthermore, ROC curves emphasize the proposed method’s effectiveness in distinguishing between positive and negative instances, which is crucial in cases with imbalanced class distributions. This demonstrates the method’s reliability and robustness for real-world applications.

**Figure 4:**
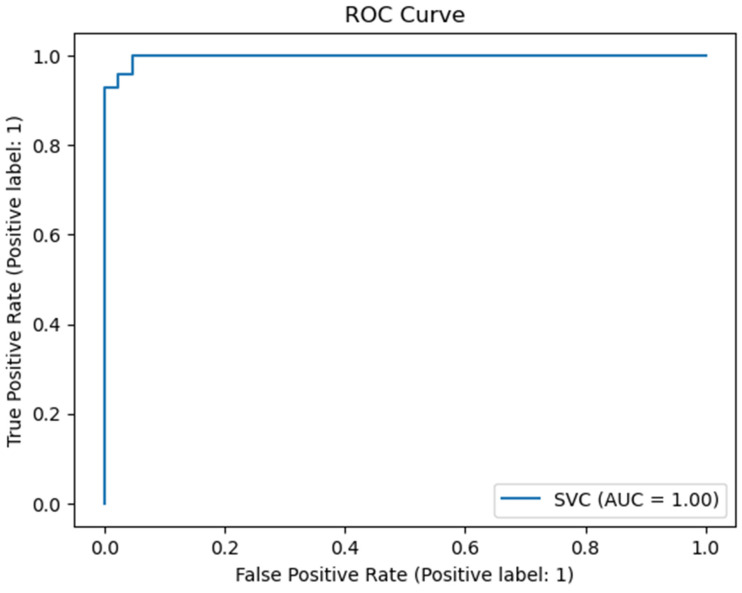
ROC curve.

Similarly, Figure 4 displays the Receiver Operating Characteristic (ROC) curve, a vital tool for evaluating classifiers. This graph demonstrates the trade-off between sensitivity (true positive rate) and the inverse of specificity (false positive rate), providing key insights into the model’s ability to distinguish between classes. A higher AUC-ROC score indicates better class differentiation. The ROC curve from the proposed method highlights its strong ability to discriminate between classes. The impressive AUC values observed in both the precision–recall (PR) and ROC curves confirm the effectiveness and reliability of the combined approach in accurately classifying cancer types. These visual tools offer strong evidence of the method’s success and its potential for clinical applications.

### 4.4. Box Plot

**Figure 5:**
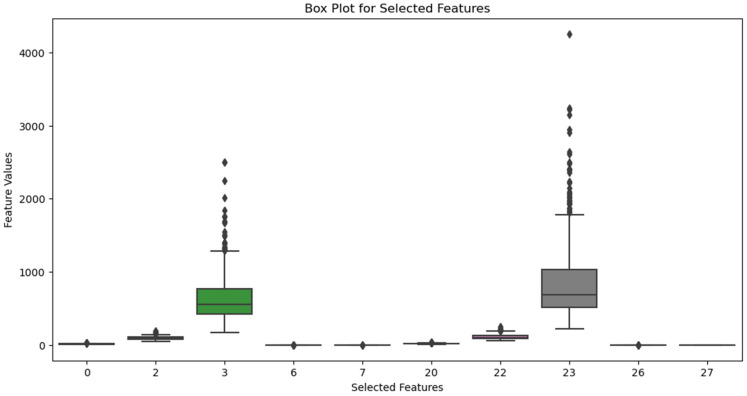
Box plot illustrating the classification accuracies of the proposed method.

Figure 5 presents a box plot analysis to evaluate the performance of the proposed Two-phase MI-PSA Gene Selection algorithm in cancer data classification. The plot shows the distribution of classification accuracies achieved by the MI-PSA method across different iterations or datasets, providing an overview of its consistency and effectiveness. Each box illustrates the interquartile range of accuracies, with the median accuracy indicated by a horizontal line inside the box. The upper and lower whiskers represent the highest and lowest accuracy values, respectively, highlighting the performance range. The box plot clearly shows that the MI-PSA approach consistently achieves superior accuracy compared to existing methods, underscoring its reliability and effectiveness in cancer data classification. This visual representation emphasizes the potential of the proposed algorithm in supporting cancer management and treatment decisions.

### 4.5. Discussion of Alternative Methods to MI, PSO, and SVM

In this section, we explore several alternative methods to the Mutual Information (MI) filtering, Particle Swarm Optimization (PSO), and Support Vector Machine (SVM) techniques used in our proposed approach. The purpose of this discussion is to evaluate other potential algorithms that can be applied to gene selection and cancer classification tasks.

### 4.6. Alternative Optimization Algorithms

While MI-based filtering and PSO have shown promising results in gene selection, there are other optimization algorithms that could potentially enhance the feature selection process.

1. Genetic Algorithms (GAs): Genetic Algorithms (GAs) are popular evolutionary algorithms inspired by natural selection. GAs use crossover, mutation, and selection operations to evolve a population of candidate solutions over successive generations. The GA approach has been widely used for feature selection due to its ability to search large and complex spaces for optimal solutions. The potential advantage of GAs over PSO lies in their robustness to local optima, as they explore the solution space by combining different solutions, which can sometimes lead to better performance. However, GAs can be computationally expensive, especially when dealing with high-dimensional data, and require careful tuning of parameters such as population size and mutation rates.
2. Ant Colony Optimization (ACO): Ant Colony Optimization (ACO) is a swarm-based optimization algorithm inspired by the foraging behavior of ants. ACO is effective for solving combinatorial optimization problems, including feature selection. Like PSO, ACO is capable of exploring a large solution space, but uses pheromone trails to guide the search for optimal solutions. ACO could potentially offer advantages in terms of discovering new solutions, but it is also computationally intensive. Furthermore, ACO’s performance can be sensitive to the choice of parameters, such as the pheromone decay rate and the number of ants.
3. Other Optimization Algorithms: Beyond GAs and ACO, other optimization algorithms like Particle Swarm Optimization (PSO), Simulated Annealing (SA), and Differential Evolution (DE) can be considered for feature selection. Each has its strengths, but PSO has been preferred in our approach due to its balance between convergence speed and solution quality in high-dimensional search spaces.

### 4.7. Alternative Classifiers

In addition to the Support Vector Machine (SVM), there are several alternative classifiers that can be considered for cancer classification tasks, which may offer improved performance or computational efficiency depending on the dataset and problem characteristics.

1. Random Forest (RF): Random Forest is an ensemble learning method that constructs multiple decision trees and outputs the mode of their predictions. RF is widely regarded for its ability to handle high-dimensional data and deal with overfitting. It is computationally less expensive compared to SVM, especially when dealing with large datasets. The ability of RF to handle non-linear relationships and interactions between features makes it a suitable alternative to SVM. However, it might be less effective than SVM in cases where the decision boundaries are highly complex or when the dataset is very sparse.
2. Deep Learning (DL): Deep learning models, particularly neural networks, have gained considerable attention in cancer classification due to their ability to learn hierarchical representations of data. Convolutional Neural Networks (CNNs) and Recurrent Neural Networks (RNNs) are powerful models that can learn complex patterns in large-scale datasets. Deep learning models could potentially offer superior performance in classification tasks, especially in cases where the relationships between features are highly non-linear. However, deep learning models tend to require much larger datasets for training and are computationally more intensive compared to traditional methods like SVM and RF. They also demand significant computational resources, making them less feasible for real-time applications or scenarios with limited data.
3. Logistic Regression (LR): Logistic Regression is a simpler and less computationally demanding model compared to SVM and RF. It performs well for binary classification problems and can be a good baseline for evaluating more complex models. However, its performance tends to degrade when there are high levels of feature interaction or non-linear relationships between features, which is common in gene expression data.
4. k-Nearest Neighbors (k-NN): The k-Nearest Neighbors algorithm is a non-parametric method that classifies new instances based on the majority vote of the k nearest data points. While it is simple to implement and computationally efficient for smaller datasets, k-NN can struggle with high-dimensional data due to the “curse of dimensionality,” where the distance between data points becomes less meaningful as the number of features increases.

### 4.8. Impact of Alternatives on Performance

The comparison of the proposed approach to alternative optimization algorithms and classifiers provides a better understanding of the strengths and weaknesses of each method. MI-based feature selection combined with PSO for optimization and SVM for classification offers a balanced approach, effectively handling high-dimensional gene expression data. However, alternative methods could be explored for specific scenarios:

- Accuracy: Deep learning models and Random Forest may achieve higher accuracy in large-scale datasets with complex patterns, especially when a large volume of training data is available. However, for smaller datasets, SVM and PSO-based approaches tend to be more reliable.
- Computational Efficiency: The proposed MI-PSO-SVM method is computationally efficient compared to deep learning models, which require extensive computational resources for training. Random Forest also tends to be more efficient than SVM in handling larger datasets, but it might not always provide the same level of accuracy, particularly when dealing with sparse or imbalanced data.
- Interpretability: Methods like Logistic Regression, Random Forest, and SVM provide a level of interpretability that is crucial in clinical settings, where understanding the importance of specific features (e.g., genes) is essential. Deep learning models, on the other hand, are often regarded as “black-box” models, making them less interpretable.

In conclusion, based on the discussions, we have summarized the key findings in Table 4. The choice of alternative optimization methods and classifiers depends on the specific characteristics of the data and the requirements of the classification task. While MI-PSO-SVM provides a strong approach for gene selection and cancer classification, exploring alternative methods may offer improvements in certain scenarios, particularly in terms of classification accuracy and computational efficiency. Table 4 shows a comparison between the proposed method and various other methods.

### 4.9. Discussion

The Two-stage MI-PSA Gene Selection algorithm demonstrates a compelling approach to tackling the challenges inherent in cancer gene selection and classification, offering significant advancements over traditional methods. By integrating Mutual Information (MI) and Particle Swarm Optimization (PSO), the MI-PSA algorithm harnesses the strengths of both feature relevance assessment and optimization-based refinement. In the first stage, MI effectively filters out genes with limited predictive power, ensuring that only those containing rich cancer-related information progress to the second stage. This initial reduction is crucial for managing the dimensionality of the data, which often poses a challenge in high-throughput genomic datasets. In the second stage, PSO fine-tunes the gene selection by iteratively searching for the optimal subset of genes, striking a balance between maximizing relevance and minimizing redundancy among selected genes. This dual-stage approach not only enhances classification performance, but also addresses the risk of overfitting by maintaining a compact, high-quality gene subset. The study’s experimental results substantiate the effectiveness of MI-PSA in cancer classification tasks. Achieving a best accuracy of 99.01% with only 19 genes, the algorithm markedly surpasses the baseline performance of MI and SVM, which achieve 93.44% and 91.26%, respectively. This significant improvement suggests that the synergy of MI and PSO in MI-PSA allows for a more refined gene selection that is better aligned with the underlying biological differences between cancer and non-cancer samples. Furthermore, MI-PSA’s performance stability—demonstrated by its consistently high average and worst-case accuracies—highlights the algorithm’s robustness, making it a reliable option for clinical applications where consistent and precise classification is paramount. Another critical consideration in gene selection and cancer classification is computational efficiency. MI-PSA’s two-stage approach effectively mitigates the computational burden often associated with high-dimensional data, especially in comparison to traditional methods that may require intensive resources for exhaustive search or purely statistical filtering. By leveraging MI as a pre-filter, MI-PSA minimizes the number of features entering the PSO stage, thus reducing computational costs and facilitating faster processing times. This efficiency is particularly advantageous in clinical contexts, where timely decision-making is essential.

Moreover, the results obtained in this study underscore the broader implications of MI-PSA in advancing precision medicine. By enabling accurate classification based on a minimal subset of highly informative genes, MI-PSA supports the development of targeted treatment strategies and early diagnostic tools. The algorithm’s ability to identify key genes that correlate strongly with cancer-related variations suggests its potential application in biomarker discovery, contributing valuable insights into cancer biology and aiding in the identification of novel therapeutic targets. In summary, the MI-PSA algorithm presents a powerful tool for gene selection in cancer research, combining the filtering precision of MI with the optimization strength of PSO to achieve superior classification accuracy. Its robustness, efficiency, and potential clinical applicability make MI-PSA a promising approach for advancing cancer diagnostics and treatment. Future research could focus on adapting MI-PSA to a broader range of cancer types and testing its scalability on larger datasets, further validating its utility in diverse biomedical contexts.

## 5. Conclusions

Classifying cancer samples is inherently complex, influenced by factors such as microarray DNA samples, cancer types, gene selection, and inherent gene subset information. In this study, we introduce an effective gene selection methodology tailored for breast cancer classification datasets. The proposed MI-PSO gene selection approach comprises two stages: Mutual Information-based gene selection followed by PSO-based refinement. Evaluation using a Support Vector Machine (SVM) classifier demonstrates the superior performance of the MI-PSO method across all datasets, achieving maximal classification accuracy. Future research could explore integrating machine learning techniques like Particle Swarm Optimization (PSO) with Fuzzy Logic and Neural Networks to further enhance microarray data classification. Such hybrid approaches hold promise for refining classification models, potentially improving accuracy while reducing the computational load.

## 6. Future Directions

Based on the promising results of the Two-stage MI-PSA Gene Selection algorithm, several future research directions can be proposed to further enhance its utility and applicability in cancer classification and other biomedical research areas.

1. Integration with Deep Learning Models:
  - Investigate the potential of integrating the MI-PSA gene selection method with deep learning architectures, such as Convolutional Neural Networks (CNNs) and Recurrent Neural Networks (RNNs). This integration could help capture complex relationships and patterns in gene expression data, leading to even higher classification accuracy and robustness.
2. Application to Other Cancer Types and Multi-Class Scenarios:
  - Extend the application of the MI-PSA algorithm to other cancer types beyond breast cancer, including multi-class classification scenarios. This would demonstrate the generalizability and effectiveness of the method across different cancer datasets with varying complexities.
3. Incorporation of Clinical and Multi-Omics Data:
  - Explore the integration of clinical data (e.g., patient demographics, clinical history) and multi-omics data (e.g., proteomics, metabolomics) with the MI-PSA approach. This holistic view could offer a more comprehensive understanding of cancer mechanisms and improve personalized treatment strategies.
4. Dynamic Adaptation of PSO Parameters:
  - Develop adaptive mechanisms for dynamically adjusting PSO parameters, such as inertia weight and acceleration coefficients, based on dataset characteristics. This could optimize the performance of the PSO algorithm in diverse gene expression datasets, enhancing gene selection efficiency and classification outcomes.
5. Real-Time Gene Selection for Personalized Medicine:
  - Investigate the feasibility of using the MI-PSA algorithm in a real-time clinical setting for personalized medicine. This could involve developing a user-friendly software tool that clinicians can use to quickly identify key genetic markers and recommend targeted therapies based on individual patient profiles.
6. Combination with Other Feature Selection Techniques:
  - Explore the combination of MI-PSA with other feature selection methods, such as Recursive Feature Elimination (RFE) or Genetic Algorithms (GAs), to create hybrid models. This could further refine gene selection and lead to even better classification performance.
7. Handling Imbalanced Datasets:
  - Develop strategies within the MI-PSA framework to effectively handle class imbalance in cancer datasets, such as incorporating synthetic data generation techniques like SMOTE (Synthetic Minority Over-sampling Technique) to improve the classification of minority classes.
8. Exploring Gene-Gene Interaction Networks:
  - Extend the MI-PSA approach to account for gene–gene interaction networks by incorporating network-based feature selection techniques. This would help in understanding the synergistic effects of gene sets and their impact on cancer progression and classification.
9. Longitudinal and Prognostic Studies:
  - Apply the MI-PSA algorithm to longitudinal cancer datasets to identify genes associated with disease progression and prognosis. This could contribute to the development of predictive models for patient outcomes and inform long-term treatment planning.
10. Benchmarking Against State-of-the-Art Methods:
  - Conduct extensive benchmarking of the MI-PSA algorithm against other state-of-the-art gene selection and classification methods using a variety of cancer datasets. This would provide a comprehensive evaluation of its strengths and potential areas for improvement.

By pursuing these directions, future research can continue to refine and expand the capabilities of the MI-PSA algorithm, contributing to more accurate cancer diagnostics and effective therapeutic strategies.

## Figures and Tables

**Table 1:** Parameter settings for the proposed method.

Stage	Parameter	Value
Filter	Approach to Selecting Genes	Mutual information
	Chosen Genes (k)	100
Wrapper	SVM (kernel)	Linear
	Size for Testing Data	20%
	Random State	42 (Seed for reproducibility)
	SVM Classifier C	Default (1.0)
	Standardization Method	Standard Scaler
	Training Size	80%

**Table 2:** Comprehensive details regarding a dataset focused on gene expression.

Data	Description	Sample	Classes	Genes
Breast Cancer [36]	Breast cancer originates in the cells of breast tissue and ranks as one of the prevalent cancer types affecting women.	97	2	24,481

**Table 3:** The classification accuracy of the breast cancer dataset using the proposed method, Mutual Information, and SVM.

Selected Genes	Accuracy								
	Proposed Method			Mutual Information			SVM		
	Best	Average	Worst	Best	Average	Worst	Best	Average	Worst
13	89.29	81.93	75.91	83.55	75.22	69.37	81.91	75.96	65.67
17	96.63	87.86	77.89	88.35	78.65	68.76	87.86	77.89	67.76
19	99.01	91.26	82.93	93.44	82.54	71.44	91.26	82.93	70.44
20	96.87	89.66	81.53	92.30	91.04	81.44	89.66	81.53	80.44
22	96.54	88.08	80.98	97.34	88.65	79.77	88.08	80.98	78.77
23	94.68	84.94	77.97	95.45	85.23	77.81	84.94	77.97	73.81
27	93.16	83.78	76.23	93.57	83.08	75.39	83.78	76.23	71.39
31	92.84	82.58	74.35	91.29	81.35	71.21	82.58	74.35	70.21
35	90.77	80.38	73.36	88.76	79.22	69.48	80.38	73.36	68.48
39	88.96	78.97	71.17	87.06	77.02	67.78	78.97	71.17	65.78
43	87.15	76.99	69.31	84.35	74.37	64.12	76.99	69.31	63.2
47	86.29	75.12	67.76	83.65	73.58	65.31	75.12	67.76	62.31

**Table 4:** Comparison based on different algorithms.

References	Algorithms	Gene No.	Accuracy
Proposed method	MI-PSO	19	99.01
[37]	GBC	19	92.19
[38]	mAnt	38	91.5
[39]	mRMR.PSO	43	90.32
[40]	GBC	15	97.38
[41]	mRMR.ABC	25	96.77
[42]	PSO	27	85.48
[43]	LGBM	12	98.6
[44]	AAElastic	29	96.4

## Data Availability

The data utilized in this research study is accessible to the public.
